# Application of transcutaneous electrical nerve stimulation in monitored anesthesia care during foraminoscopy: a randomized double-blind controlled trial

**DOI:** 10.3389/fmed.2025.1622657

**Published:** 2025-06-26

**Authors:** Shuhui Sun, Lei Zhang, Dongdong Yu, Li Jiang, Ge Yang, Long Zhang, Yu Tian, Chunxiao Xu, Dong Zhang

**Affiliations:** ^1^Department of Anesthesiology, Hebei General Hospital, Shijiazhuang, China; ^2^Graduate School of Hebei Medical University, Shijiazhuang, China; ^3^Department of Orthopedics, Hebei General Hospital, Shijiazhuang, China; ^4^Graduate School of Hebei North University, Zhangjiakou, China

**Keywords:** transcutaneous electrical nerve stimulation, trans-auricular vagus nerve stimulation, Visual Analogue Scale, heart rate variability, foraminoscopy

## Abstract

**Background:**

As minimally invasive surgical techniques have evolved, foraminoscopy has emerged as a predominant surgical procedure. This technique utilizes the intervertebral foramen as the primary surgical pathway and addresses lumbar intervertebral disk issues through endoscopic intervention. However, since the surgery is performed under local anesthesia, achieving satisfactory anesthesia poses significant challenges. This investigation sought to assess the efficacy and comparative differences between two non-pharmacological analgesic approaches: transcutaneous electrical nerve stimulation (TENS) and trans-auricular vagus nerve stimulation (taVNS), in the context of monitored anesthesia care (MAC) during foraminoscopy.

**Methods:**

This investigation adopted a randomized, double-blind, controlled trial design. The participants were randomly divided into the TENS group and the taVNS group. Each group was further divided into three stimulation modes: continuous wave, intermittent wave, and dense–sparse wave. In each group, electrical stimulation was applied 30 min prior to surgery. The main parameters to be evaluated included the patients’ Visual Analogue Scale (VAS) scores, heart rate variability (HRV), bispectral index (BIS), Modified Observer’s Assessment of Alertness/Sedation Scale (MOAA/S) and the supplemental use of oliceridine fumarate during the surgery.

**Discussion:**

This study explores the effect of non-pharmacological electrical stimulation techniques in MAC during foraminoscopy through a randomized controlled trial. If proven effective, these techniques could provide viable non-pharmacological analgesic alternatives for foraminoscopy procedures. Additionally, by comparing different waveform stimulations, the electrical stimulation parameters can be further optimized.

## 1 Introduction

Foraminoscopy represents a minimally invasive spinal surgical procedure. In the treatment of lumbar intervertebral disks, it mainly adopts percutaneous endoscopic lumbar discectomy, which has the advantages of minimal trauma, rapid recovery, clearer surgical field, and enhanced safety (1). Performing foraminoscopy under local anesthesia has the merits of low anesthetic risk, higher precision, and the ability for patients to communicate with doctors during the operation (2). However, since local anesthesia may not completely eliminate discomfort during the surgery, it can increase patients’ nervousness and discomfort. Therefore, there is room for optimization in the anesthesia method. In response to this, different modes of electrical stimulation are considered as adjuvant analgesia in the anesthesia plan.

The technique is termed MAC when an anesthesia practitioner monitors a patient receiving local anesthesia and/or administers sedative-analgesic medications to patients undergoing diagnostic or therapeutic procedures (3). MAC administered in conjunction with local anesthesia provides patients with moderate sedation and analgesia. This effectively alleviates anxiety and intraoperative discomfort, enhances patient cooperation, and offers advantages over general anesthesia, including reduced dosage of sedative and analgesic medications and faster postoperative recovery (4). Currently, MAC serves as the preferred technique for various procedures both inside and outside the operating room, including ophthalmic surgery, otolaryngologic (ENT) surgery, pain procedures, and endoscopy. However, due to the frequent absence of airway protection during MAC, it is crucial to avoid excessive sedation which can lead to central respiratory depression.

The auricular branch of the vagus nerve (ABVN) is the vagus nerve’s only superficial branch, distributed to the auricle, auricular concha, tragus, and other areas. A meta-analysis involving 17 RCTs indicates that 75% of localized auricular acupuncture stimulation primarily targets regions innervated by the ABVN (5). TaVNS exerts modulatory effects on the brain by stimulating the ABVN, thereby influencing the central nervous system (6). In recent years, significant progress has been made in the treatment of chronic pain, epilepsy, and severe depression (7). It has been shown that electrical stimulation of the vagus nerve in the patient’s ear area reduces the frequency of seizures in drug-resistant epilepsy (8), and is thought to have a significant effect on autonomic modulation, which can be effective in controlling pain and sedative effects. In terms of neurophysiology, taVNS activates brainstem centers by stimulating the afferent fibers of the ABVN, thereby enhancing efferent parasympathetic nerve activity. This process increases acetylcholine release onto the sinoatrial node of the heart, leading to reduced heart rate and increased vagally-mediated HRV (9). The precise mechanisms and effects of taVNS are not yet fully understood. Preliminary research suggests that taVNS may stimulate the cholinergic anti-inflammatory pathway, resulting in reduced production of pro-inflammatory cytokines (e.g., IL-1β, IL-6), thereby exerting anti-inflammatory effects (10). It may also modulate noradrenergic activity in the locus coeruleus (LC), consequently influencing the body’s autonomic nervous system (11). Furthermore, no consensus has been reached regarding the optimal stimulation duration, the most effective sites for stimulation, or the ideal stimulation parameters (12).

Peripheral nerve stimulation (PNS) modulates pain signaling by directly stimulating peripheral nerves in the limbs, primarily affecting the peripheral nervous system. TENS based on the Gate Control Theory, utilizes high-frequency, low-intensity stimulation to activate large-diameter Aβ afferent fibers. This induces segmental inhibition of nociceptive signal transmission at the level of the spinal cord dorsal horn (13). Contemporary applications frequently combine TENS with traditional Chinese acupuncture for acupoint electrical stimulation in pain management, proving effective for various chronic pain conditions. For example, Mu et al. (14) demonstrated that electrical stimulation at the Zusanli acupoint can relieve abdominal pain after gastrointestinal surgery; Zhang et al. (15) analyzed relevant articles and concluded that percutaneous acupoint electrical stimulation can improve the postoperative analgesic effect of patients under general anesthesia and reduce the incidence of postoperative nausea and vomiting. Simultaneously, (16) studies in lumbar orthopedic surgery have indicated that electrical stimulation applied at the Neiguan (PC6) and Hegu (LI4) acupoints, when used as an adjunct to general anesthesia, effectively maintains heart rate stability and reduces the surgical stress response in patients undergoing open posterior lumbar spine surgery. However, more high–quality research is urgently needed to further explore its application in monitored anesthesia and provide patients with more personalized and comfortable analgesic plans.

Oliceridine fumarate is a novel G protein-biased ligand μ-opioid receptor agonist. Approved by the FDA in 2020, it represents the first intravenously administered opioid medication approved by the agency in decades. Relevant studies have demonstrated its potent analgesic efficacy, with onset and duration of action comparable to morphine (17–19).

Compared to traditional opioids such as morphine and fentanyl—which, while effective for pain relief—are frequently limited by opioid-related adverse events (AEs) including respiratory depression, nausea, vomiting, ileus, and excessive sedation (20), clinical studies indicate improved safety profiles for oliceridine versus morphine (21, 22). Consequently, it holds promise as a new alternative for analgesia during MAC.

This study investigates the combined application of electrical stimulation and oliceridine fumarate injection into MAC, studies the differences in the effects of TENS and taVNS on patients’ heart rate variability, Visual Analogue Scale scores, and bispectral index, explores the differences in the efficacy of different waveforms in monitored anesthesia, expands the application scope of transcutaneous electrical stimulation, further optimizes the electrical stimulation parameters, and simultaneously explores the reduction of the dosage of oliceridine fumarate injection under different stimulations to improve the effect of monitored anesthesia technology, thereby providing patients with more comfortable and personalized plans.

## 2 Materials and methods

### 2.1 Study design

#### 2.1.1 Trial design

This experiment is designed as a randomized controlled clinical trial (RCT). The research subjects are surgical patients undergoing foraminoscopy under monitored anesthesia in Hebei General Hospital. The effects of TENS and taVNS are compared through random grouping. This research protocol is written in accordance with the recommendations of the Standard Protocol Items: Recommendations for Interventional Trials (SPIRIT).

#### 2.1.2 Study setting

This study will be conducted in the Department of Anesthesiology, Hebei General Hospital, China.

#### 2.1.3 Participants

All participants will be strictly screened according to the inclusion and exclusion criteria. Researchers will introduce the experimental procedures, consent forms, and the established plans in detail to the subjects. Eligible volunteers will be invited to participate in the study. After signing the informed consent form, they will undergo a baseline assessment.

#### 2.1.4 Eligibility criteria

##### 2.1.4.1 Inclusion criteria

• Aged 18–65 years old, conscious and with civil capacity, and having no contraindications to surgery and anesthesia.
• Signed the informed consent form.

##### 2.1.4.2 Exclusion criteria

• Patients with severe respiratory depression, gastrointestinal obstruction, severe arrhythmia, severe liver insufficiency.
• Patients who experience severe adverse reactions during the operation and have the operation terminated or the anesthesia method changed.
• Patients with neurological diseases such as epilepsy or a history of epilepsy.
• Patients known to be allergic to oliceridine.
• Patients with skin infections or damage at the stimulation site.

##### 2.1.4.3 Withdrawal criteria

• Deviation from the assigned anesthesia protocol.
• Occurrence of any serious adverse event.
• Intraoperative administration of heart rate-altering medications (e.g., atropine).
• Failure to complete all required monitoring procedures.

#### 2.1.5 Who will take informed consent?

Data collection will only start after the subjects sign the informed consent form, and the assessment personnel will explain this.

#### 2.1.6 Additional consent provisions for collection and use of participant data and biological specimens

Biological specimens will not be collected in this experiment.

### 2.2 Randomization and blinding

Patients are divided into two groups (TENS group and taVNS group) through a random number table generated by a computer. Each group is designed with a control group and three stimulation subgroups. A double–blind design is adopted, and neither the interveners (personnel implementing electrical stimulation interventions) nor the patients are aware of the grouping situation. They do not have access to the group allocation sequence, are not involved in randomization procedures, and do not participate in outcome assessments. To ensure effective blinding, all electrode pads will be covered with identical opaque adhesive membranes, with only the conductive contact point exposed. Additionally, electrode pads will be applied to both the auricular and acupoint regions in all participants, regardless of group allocation. As a result, the interveners will not know which site is actively receiving stimulation.

### 2.3 Sample size calculation

This study is a randomized controlled trial aiming to compare the effects of TENS and taVNS in foraminoscopy. According to relevant research, with the VAS as the primary outcome indicator, the standard deviation of taVNS is 2.5, and that of TENS is 3.0. Calculated using PASS software, the minimum sample size required for each group is 121. Considering a dropout rate of 10%–15%, to ensure the statistical power and the reliability of the results, the final sample size of each group is adjusted to 136 people. Each subgroup was allocated 32 participants, and a total of 272 participants are recruited.

### 2.4 Intervention measures

#### 2.4.1 Explanation for the choice of comparators

Previous studies have shown that both TENS and taVNS have certain effects on pain regulation. However, there are few studies comparing the differences in the efficacy of the two, and the electrical stimulation parameters have not been made public. Therefore, this study aims to compare the differences in the efficacy of the two and obtain the appropriate electrical stimulation parameters for foraminoscopy.

#### 2.4.2 Intervention description

Based on a systematic review and related studies (23), each group receives 30-min corresponding stimulation or placebo intervention before surgery. The current intensity is adjusted to the maximum tolerance of the patient, the pulse width is fixed at 200 μs, and the frequency is fixed at 30 Hz. When patients with severe pain cannot tolerate it, oliceridine fumarate injection is supplemented to the subjects. The supplementary standard is: a single dose of 0.35 mg–0.50 mg, intravenous injection, and the dosing interval is not less than 6 min.

• TENS Group: Electrodes are placed at the bilateral Neiguan and Hegu acupoints of the subjects. Hegu (LI4): Located on the dorsum of the hand, between the 1st and 2nd metacarpal bones, at the midpoint of the radial side of the second metacarpal bone. Neiguan (PC6): Located on the palmar aspect of the forearm, 2 cun proximal to the transverse wrist crease, between the tendons of the palmaris longus and the flexor carpi radialis muscles.
• TaVNS Group: Electrodes are placed at the bilateral tragus of the subjects.

Each stimulation group was further subdivided into following stimulation modes:

• Continuous Wave Group: Given 30-min continuous electrical stimulation.
• Intermittent Wave Group: Subjects receive 30-min intermittent electrical stimulation (stimulate once every 15 s, with an interval of 5 s).
• Dense-Sparse Wave Group: Subjects receive 30-min variable–frequency electrical stimulation (sparse wave 6 Hz, dense wave 30 Hz).
• Control Group: Electrode patches are placed but not energized, making the patients psychologically believe that they have received stimulation.

**FIGURE 1:**
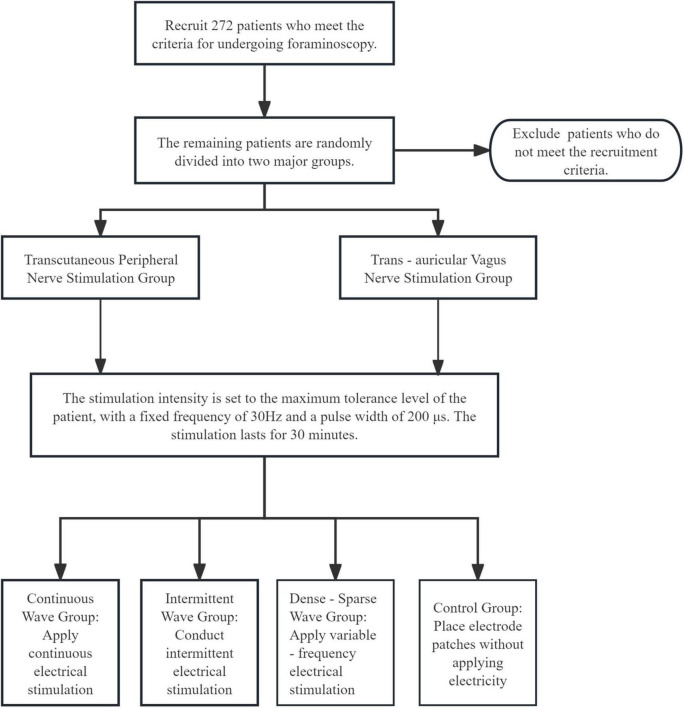
Flowchart of experimental intervention grouping.

The experimental process is shown in Figure 1.

##### 2.4.2.1 Observation indicators

Record the changes of corresponding indicators at different time points:

• Time 1: 5 min after the patient enters the operating room, before electrical stimulation.
• Time 2: Before the operation, 30 min after the electrical stimulation upon entering the room.
• Time 3: Immediately at the start of the operation.
• Time 4: 0.5 h after the start of the operation.
• Time 5: 1.0 h after the start of the operation.
• Time 6: 1.5 h after the start of the operation.
• Time 7: 2.0 h after the start of the operation.
• Time 8: At the end of the operation.

###### 2.4.2.1.1 Criteria for discontinuing or modifying allocated interventions

If a subject withdraws from the experiment midway or is not satisfied with the analgesic effect, the recent data will be calculated for analysis according to the intention–to–treat principle. Conventional drugs will be used for analgesia and sedation management, and the reason for withdrawing from the study will be recorded.

###### 2.4.2.1.2 Strategies to improve adherence to interventions

Before the surgery, explain the exact effects of the electrical stimulation–related therapies to the patients who have signed the informed consent form to reduce their preoperative fear and tension.

###### 2.4.2.1.3 Relevant concomitant care permitted or prohibited during the trial

During the entire trial, subjects are required not to use any other analgesic measures or drugs, as this may affect the outcome.

### 2.5 Outcomes

#### 2.5.1 Primary observation indicators

• Bispectral index (BIS) to assess the depth of anesthesia.
• Modified Observer’s Assessment of Alertness/Sedation Scale (MOAA/S) and Ramsay sedation scale to assess the sedation level of patients.
• Visual Analogue Scale (VAS) to assess the pain level of patients.

Heart rate variability (HRV) is the variability of cycle-to-cycle differences in heart rate, which is mainly controlled by the autonomic nerves of the heart, and has been widely recognized as a valid indicator for assessing autonomic function. Heart rate is monitored and recorded using a three-lead ECG. We can use time-domain, frequency-domain, and non-linear measures to characterize HRV over 24 h, short-term or transient and ultrashort-term (24). Among the frequency-domain indices: the high-frequency component reflects parasympathetic regulation of heart rate (25), whereas the low-frequency component reflects sympathetic and parasympathetic actions (26). The ratio of low-frequency (LF) to high-frequency (HF) in HRV can be used as an indicator of cardiac autonomic balance, i.e., a decrease in the LF/HF ratio indicates a shift in cardiac autonomic balance toward parasympathetic dominance, which leads to an improvement in HRV (27). Time-domain metrics quantify the amount of change in interbeat interval measurements. Time domain analysis was recorded and analyzed from: AVNN, SDNN, RMSSD, SDANN, and NN50. For non-linear measurements the Poincaré plot was analyzed by fitting an ellipse. This resulted in three non-linear measurements, i.e., S, SD1, and SD2.

#### 2.5.2 Secondary observation indicators

• Vital signs such as blood pressure, heart rate, pulse oximetry saturation, blood glucose, and perfusion index.
• Adverse events including local skin reactions (redness and swelling), transient neurological symptoms (headache and dizziness), gastrointestinal disturbances (nausea and vomiting), and cardiovascular effects (bradycardia and hypotension).

#### 2.5.3 Participant timeline

An overview of the study procedures is shown in Table 1.

**TABLE 1:** Schedule of enrolment, interventions, and assessments.

	Study period										
	Enrollment	Allocation	Post-allocation								Close-out
Timepoint	−T1	T0	T1	T2	T3	T4	T5	T6	T7	T8	Tx
Enrollment	X										
Eligibility screen	X										
Informed consent	X										
Allocation		X									
**Intervention**											
TENS				X							
TaVNS				X							
**Assessments**											
VAS			X	X	X	X	X	X	X	X	
BIS			X	X	X	X	X	X	X	X	
MOAA/S			X	X	X	X	X	X	X	X	
Ramsay			X	X	X	X	X	X	X	X	
HRV			X	X	X	X	X	X	X	X	
Baseline			X								

## 3 Statistical analysis methods

### 3.1 Statistical methods for primary and secondary outcomes

This study will use SPSS 20.0 and PhysioZoo software for statistical analysis. The significance level of all statistical tests is set at α = 0.05, and two-sided tests are performed. For the differences in different intraoperative indicators such as pain score VAS, anesthesia depth BIS, and heart rate variability HRV between the trans-auricular vagus nerve stimulation group (taVNS group) and the transcutaneous peripheral nerve stimulation group (TENS group), an independent-samples *t*-test is first performed. If the data do not meet the normal distribution, the non-parametric test method Mann–Whitney U test will be used for comparison between the two groups. For the comparison between different subgroups (different stimulation modes) within each group, one-way analysis of variance will be performed to compare the differences in various indicators. If the data do not meet the assumptions of analysis of variance, the Kruskal–Wallis H test will be used as a non-parametric alternative method. At the same time, for multiple-measurement data, repeated-measures analysis of variance (Repeated Measures ANOVA) will be used to compare the changes of indicators at different time points within the same group. To further evaluate the impact of different influencing factors (such as stimulation mode) on intraoperative analgesia and sedation effects, multiple linear regression analysis will be used to help identify their relationships with various indicators.

### 3.2 Methods for additional analyses

There are currently no plans for additional data analysis.

### 3.3 Methods in analysis to handle protocol non–adherence and any statistical methods to handle missing data

Intention-to-treat analysis will be used, and missing data will be appropriately processed in accordance with established guidelines.

### 3.4 Interim analyses

No interim analysis is planned.

### 3.5 Expected results

The expected research results are that taVNS may exert sedative effects and potentially reduce the use of adjuvant analgesic drugs and has a positive effect on the relevant indicators of heart rate variability, which is a new and effective anesthesia-assisting method during foraminoscopy. The specific manifestations are as follows: 1. The taVNS group has a more obvious analgesic effect (the VAS score increases more significantly), a more obvious impact on the relevant indicators of HRV (the standard deviation of heart rate increases, RMSSD and SDNN decrease, and LF increases), a lower BIS value, and a significant reduction in the additional amount of oliceridine fumarate for patients compared with the TENS group. This indicates that the regulatory role of the vagus nerve plays an important role in pain regulation, and taVNS enhances the activity of the parasympathetic nerve, making the depth of anesthesia more stable; 2. TENS has a certain analgesic effect, but its impact on HRV is not significant; 3. Among different stimulation modes, the dense-sparse wave group (sparse wave 6 Hz, dense wave 30 Hz) has more obvious changes in HRV, VAS, BIS, and sedation under the parameters of 30 Hz and 200-pulse width. At the same time, by using the Huatuo brand electronic diagnosis and treatment instrument SDZ-IIB, a more effective and safer stimulation mode for the sedative and analgesic effects of patients can be obtained, providing more simple and effective treatment guidance for patients.

## 4 Discussion

HRV refers to the variation in time intervals between consecutive heartbeats and is mainly controlled by the cardiac autonomic nerves. It has been widely recognized as an effective indicator for evaluating autonomic nerve function.

The application of TENS and taVNS represents an emerging analgesic, sedative, and neuromodulatory strategy during foraminoscopy. These two non-pharmacological, minimally invasive modulation techniques are considered potentially beneficial for optimizing perioperative management, though their effects require cautious evaluation. Both modalities deliver electrical currents via stimulators, constituting a primary source of electromagnetic interference. Consequently, they are contraindicated in patients with implantable cardioverter defibrillators (ICDs), with interference levels demonstrating significant correlation to stimulation parameters that necessitate strict control (28). Additionally, substantial challenges exist in parameter selection due to individual variability in:neural sensitivity to stimulation, stimulation tolerance thresholds, psychological factors. Electrode placement for TENS should avoid surgical fields to prevent contamination. Notably, high-quality clinical studies investigating TENS and taVNS applications during MAC for transforaminal endoscopy remain limited. This study aims to provide novel clinical data in this domain.

Some studies have shown that a higher HRV is usually associated with a healthy state, stronger adaptability, and a lower disease risk (29); while a lower HRV is related to anxiety, stress, cardiovascular diseases, etc (24). In addition, Billman (30) found that HRV is also related to the body’s stress response, and the cardiac parasympathetic nerve activity is the main part of HRV. In this paper, the regulation of cardiac vagus nerve and parasympathetic nerve is quantitatively and dynamically evaluated by analyzing the frequency domain, time domain, and non-linear analysis methods of HRV. The VAS is a commonly used subjective assessment tool, which is widely used for various subjective experiences such as pain and anxiety. By using HRV and VAS together, this study comprehensively evaluates the intervention effect from both physiological and psychological dimensions. The BIS value is an important tool for assessing the depth of anesthesia during the operation. Together with the sedation scale (MOAA/S) and Ramsay sedation scale, the intraoperative state of patients can be evaluated in detail to ensure efficient evaluation.

This study explored the differences in the curative effects of TENS and taVNS in monitored anesthesia during foraminoscopy. The results of the study are expected to show that transcutaneous electrical nerve stimulation not only has a certain effect on sedation and analgesia, but also can reduce the use of auxiliary analgesic drugs and has a positive effect on the indexes of HRV, which is a new and effective anesthesia auxiliary modality in intervertebral foraminoscopy. The specific performance is as follows: 1. The taVNS group has a more obvious analgesic effect than percutaneous peripheral nerve stimulation, a more obvious effect on the relevant indexes of HRV (increase in the standard deviation of heart rate, increase in RMSSD, decrease in SDNN, and increase in LF), a significant decrease in the LF/HF ratio by taVNS, a change in the autonomic balance of the heart to a parasympathetic/vagal dominance, a significant decrease in the patient’s response to oxalidine fumarate, and a significant decrease in the amount of additional drugs, which can be shown to be a new and effective anesthetic aid in intervertebral foraminoscopy. The additional amount was significantly reduced, which can indicate that the modulation of vagus nerve plays an important role in pain modulation, and the taVNS enhanced the activity of parasympathetic nerves and stabilized the depth of anesthesia; 2. TENS had a certain analgesic effect, but its effect in HRV was not significant; 3. In different stimulation modes, the dense-sparse wave group (sparse wave 6 Hz-dense wave 30 Hz). Among the different stimulation modes, the sparse-dense wave group (sparse wave 6 Hz-dense wave 30 Hz) had more obvious changes in HRV, VAS, BIS, and sedation under the parameters of 30 Hz and 200 pulse width. At the same time, by using the electronic diagnostic instrument SDZ-IIB, it is possible to derive more effective and safer stimulation modes for patients’ sedation and analgesia, and to provide patients with simpler and more effective therapeutic guidance.

Previous studies in related fields have shown that taVNS has a positive impact on patients’ sedation and analgesia, heart rate variability, etc., and at the same time reduces the use of opioids (31). TENS may have a more obvious local analgesic effect, but its impact on heart rate variability is relatively limited. Parseliunas et al. (32) studied that TENS is of great significance in relieving postoperative pain after inguinal hernia repair, which is consistent with the expected results of this study. However, some studies have different conclusions. Gibson et al. (33) found that there is not enough evidence for the therapeutic effect of TENS on neuropathic pain in adults. These differences may be related to differences in experimental design, electrical stimulation parameters, sample characteristics, etc.

### 4.1 Research limitations

Although this experiment provides valuable insights into the anesthesia-assisting technology during foraminoscopy, there are also some limitations. The sample size of each group in this experiment is relatively small, and it is limited to the age group of 18–65 years old with certain limitations on the health status of the subjects. Therefore, the applicability of the results is questionable. Secondly, this experiment only monitors the changes in the intraoperative state of patients and does not conduct long-term postoperative follow-up. Therefore, it is impossible to evaluate the impact of electrical stimulation on postoperative recovery and long-term effects.

Currently, there are relevant research plans proposing the impact of TENS combined with TaVNS stimulation on the pain threshold of subjects (34). By analyzing the differences in the analgesic efficacy of the two, a more rigorous design in the combined plan can be carried out, which may be an important direction in the field of electrical stimulation and pain in the future. Meanwhile taVNS has been shown to be therapeutically useful for underlying heart failure as well as atrial fibrillation (35) with positive clinical results. Its mechanism of action is likely to be related to its effects on the autonomic nervous system.

## References

[1] Pan M Li Q Li S Mao H Meng B Zhou F Percutaneous endoscopic lumbar discectomy: Indications and complications. *Pain Phys.* (2020) 23:49–56. 10.36076/ppj.2020/23/49 32013278

[2] Ruan W Feng F Liu Z Xie J Cai L Ping A. Comparison of percutaneous endoscopic lumbar discectomy versus open lumbar microdiscectomy for lumbar disc herniation: A meta-analysis. *Int J Surg*. (2016) 31:86–92. 10.1016/j.ijsu.2016.05.061 27260312

[3] Murphy F. The changing role of monitored anesthesia care in the ambulatory setting. *Anesth Analg*. (1998) 86:1335–6. 10.1097/00000539-199806000-00045 9620534

[4] Vojteer N Sagawe V Stauffer J Schroeder M Hillebrecht H. LiB12PC, the first boron-rich metal boride with phosphorus–synthesis, crystal structure, hardness, spectroscopic investigations. *Chemistry*. (2016) 17:3128–35. 10.1002/chem.201002968 21308812

[5] Usichenko T Hacker H Lotze M. Transcutaneous auricular vagal nerve stimulation (taVNS) might be a mechanism behind the analgesic effects of auricular acupuncture. *Brain Stimul*. (2017) 10:1042–4. 10.1016/j.brs.2017.07.013 28803834

[6] Machetanz K Berelidze L Guggenberger R Gharabaghi A. Transcutaneous auricular vagus nerve stimulation and heart rate variability: Analysis of parameters and targets. *Auton Neurosci*. (2021) 236:102894. 10.1016/j.autneu.2021.102894 34662844

[7] Johnson R Wilson C. A review of vagus nerve stimulation as a therapeutic intervention. *J Inflamm Res*. (2018) 11:203–13. 10.2147/JIR.S163248 29844694 PMC5961632

[8] Bauer S Baier H Baumgartner C Bohlmann K Fauser S Graf W Transcutaneous Vagus Nerve Stimulation (tVNS) for treatment of drug-resistant epilepsy: A randomized, double-blind clinical trial (cMPsE02). *Brain Stimul*. (2016) 9:356–63. 10.1016/j.brs.2015.11.003 27033012

[9] Thal S Shityakov S Salvador E Förster C. Heart rate variability, microvascular dysfunction, and inflammation: Exploring the potential of taVNS in managing heart failure in type 2 diabetes mellitus. *Biomolecules*. (2025) 15:499. 10.3390/biom15040499 40305215 PMC12024555

[10] Sorski L Gidron Y. The vagal nerve, inflammation, and diabetes-A holy triangle. *Cells*. (2023) 12:1632. 10.3390/cells12121632 37371102 PMC10297454

[11] Bömmer T Schmidt L Meier K Kricheldorff J Stecher H Herrmann C Impact of stimulation duration in taVNS-exploring multiple physiological and cognitive outcomes. *Brain Sci*. (2024) 14:875. 10.3390/brainsci14090875 39335371 PMC11430400

[12] Ghani S Vilensky J Turner B Tubbs R Loukas M. Meta-analysis of vagus nerve stimulation treatment for epilepsy: Correlation between device setting parameters and acute response. *Childs Nerv Syst*. (2015) 31:2291–304. 10.1007/s00381-015-2921-1 26493055

[13] Peng W Tang Z Zhang F Li H Kong Y Iannetti G Neurobiological mechanisms of TENS-induced analgesia. *Neuroimage*. (2019) 195:396–408. 10.1016/j.neuroimage.2019.03.077 30946953 PMC6547049

[14] Mu L Gao H Zhao ML Ren HF Ma HS. [Effect of transcutaneous electrical acupoint stimulation on recovery of gastrointestinal function after cesarean section]. *Zhongguo Zhen Jiu.* (2019) 39:259–62. 10.13703/j.0255-2930.2019.03.010 30942011

[15] Zhang M Zhang H Li P Li J. Effect of transcutaneous electrical acupoint stimulation on the quality of postoperative recovery: A meta-analysis. *BMC Anesthesiol*. (2024) 24:104. 10.1186/s12871-024-02483-z 38504188 PMC10949587

[16] Xu ZQ Ding FF Zhang J Xue Y Hou HJ Xue JJ. [Effects of transcutaneous electrical acupoint stimulation at different times assisted general anesthesia on stress response of patients undergoing open posterior lumbar surgery]. *Zhen Ci Yan Jiu.* (2023) 48:481–7. 10.13702/j.1000-0607.20211314 37247862

[17] Dahan A van Dam C Niesters M van Velzen M Fossler M Demitrack M Benefit and risk evaluation of biased μ-receptor agonist oliceridine versus morphine. *Anesthesiology*. (2020) 133:559–68. 10.1097/ALN.0000000000003441 32788558

[18] Simons P van der Schrier R van Lemmen M Jansen S Kuijpers K van Velzen M Respiratory effects of biased ligand oliceridine in older volunteers: A pharmacokinetic-pharmacodynamic comparison with morphine. *Anesthesiology*. (2023) 138:249–63. 10.1097/ALN.0000000000004473 36538359

[19] Moss L Hijma H Demitrack M Kim J Groeneveld G van Velzen M Neurocognitive effect of biased μ-opioid receptor agonist oliceridine, a utility function analysis and comparison with morphine. *Anesthesiology*. (2023) 139:746–56. 10.1097/ALN.0000000000004758 37656771

[20] Paul A Smith C Rahmatullah M Nissapatorn V Wilairatana P Spetea M Opioid analgesia and opioid-induced adverse effects: A review. *Pharmaceuticals*. (2021) 14:1091. 10.3390/ph14111091 34832873 PMC8620360

[21] DeWire S Yamashita D Rominger D Liu G Cowan C Graczyk T A G protein-biased ligand at the μ-opioid receptor is potently analgesic with reduced gastrointestinal and respiratory dysfunction compared with morphine. *J Pharmacol Exp Ther*. (2013) 344:708–17. 10.1124/jpet.112.201616 23300227

[22] Stahl E Bohn L. Low intrinsic efficacy alone cannot explain the improved side effect profiles of new opioid agonists. *Biochemistry*. (2022) 61:1923–35. 10.1021/acs.biochem.1c00466 34468132 PMC8885792

[23] Patel A Weber V Gourine A Ackland G. The potential for autonomic neuromodulation to reduce perioperative complications and pain: A systematic review and meta-analysis. *Br J Anaesth*. (2022) 128:135–49. 10.1016/j.bja.2021.08.037 34801224 PMC8787777

[24] Shaffer F Ginsberg J. An overview of heart rate variability metrics and norms. *Front Public Health*. (2017) 5:258. 10.3389/fpubh.2017.00258 29034226 PMC5624990

[25] Chapleau M Sabharwal R. Methods of assessing vagus nerve activity and reflexes. *Heart Fail Rev*. (2011) 16:109–27. 10.1007/s10741-010-9174-6 20577901 PMC4322860

[26] Clancy J Mary D Witte K Greenwood J Deuchars S Deuchars J. Non-invasive vagus nerve stimulation in healthy humans reduces sympathetic nerve activity. *Brain Stimul*. (2014) 7:871–7. 10.1016/j.brs.2014.07.031 25164906

[27] Heart rate variability. Standards of measurement, physiological interpretation, and clinical use. Task Force of the European Society of Cardiology and the North American Society of Pacing and Electrophysiology. *Eur Heart J.* (1996) 17:354–81. 10.1093/oxfordjournals.eurheartj.a014868 8737210

[28] Suhail Arain S Cretnik A Huemer M Attanasio P Nagel P Landmesser U Risk of occurrence of electromagnetic interference from the application of transcutaneous electrical nerve stimulation on the sensing function of implantable defibrillators. *Europace*. (2023) 25:euad206. 10.1093/europace/euad206 37487241 PMC10365842

[29] Thayer J Lane R. The role of vagal function in the risk for cardiovascular disease and mortality. *Biol Psychol*. (2007) 74:224–42. 10.1016/j.biopsycho.2005.11.013 17182165

[30] Billman G. The effect of heart rate on the heart rate variability response to autonomic interventions. *Front Physiol*. (2013) 4:222. 10.3389/fphys.2013.00222 23986716 PMC3752439

[31] Patel A Bibawy P Althonayan J Majeed Z Gan W Abbott T Effect of transauricular nerve stimulation on perioperative pain: A single-blind, analyser-masked, randomised controlled trial. *Br J Anaesth*. (2023) 130:468–76. 10.1016/j.bja.2022.12.025 36822987 PMC10080471

[32] Parseliunas A Paskauskas S Kubiliute E Vaitekunas J Venskutonis D. Transcutaneous electric nerve stimulation reduces acute postoperative pain and analgesic use after open inguinal hernia surgery: A randomized, double-blind, placebo-controlled trial. *J Pain*. (2021) 22:533–44. 10.1016/j.jpain.2020.11.006 33309784

[33] Gibson W Wand B O’Connell N. Transcutaneous electrical nerve stimulation (TENS) for neuropathic pain in adults. *Cochrane Database Syst Rev*. (2017) 9:CD011976. 10.1002/14651858.CD011976.pub2 28905362 PMC6426434

[34] Liebano R Awad N Bellino C Bray K Rosentrater H Roy J The combined effect of transcutaneous electrical nerve stimulation and transcutaneous auricular vagus nerve stimulation on pressure and heat pain thresholds in pain-free subjects: A randomized cross-over trial. *Trials*. (2024) 25:516. 10.1186/s13063-024-08352-x 39085951 PMC11290061

[35] De Ferrari G Crijns H Borggrefe M Milasinovic G Smid J Zabel M Chronic vagus nerve stimulation: A new and promising therapeutic approach for chronic heart failure. *Eur Heart J*. (2011) 32:847–55. 10.1093/eurheartj/ehq391 21030409

